# Plasmon Resonance in a System of Bi Nanoparticles Embedded into (Al,Ga)As Matrix

**DOI:** 10.3390/nano14010109

**Published:** 2024-01-02

**Authors:** Vitalii I. Ushanov, Sergey V. Eremeev, Vyacheslav M. Silkin, Vladimir V. Chaldyshev

**Affiliations:** 1 Ioffe Institute, 26 Politekhnicheskaya Str., 194021 Saint Petersburg, Russia; ushanovvi@mail.ioffe.ru; 2 Institute of Strength Physics and Materials Science, Siberian Branch, Russian Academy of Sciences, 634055 Tomsk, Russia; eremeev@ispms.tsc.ru; 3 Saint Petersburg State University, 199034 Saint Petersburg, Russia; 4 Departamento de Polímeros y Materiales Avanzados: Física, Química y Tecnología, Facultad de Ciencias Químicas, Universidad del País Vasco (UPV-EHU), Apdo. 1072, E-20080 San Sebastián, Basque Country, Spain; 5 Donostia International Physics Center (DIPC), Paseo de Manuel Lardizabal 4, E-20018 San Sebastián, Basque Country, Spain; 6 Ikerbasque, Basque Foundation for Science, E-48011 Bilbao, Basque Country, Spain

**Keywords:** metal-semiconductor metamaterials, metal nanoparticles, dielectric function, optical absorption, localized plasmon resonance

## Abstract

We reveal the feasibility of the localized surface plasmon resonance in a system of Bi nanoparticles embedded into an Al${}_{x}$Ga${}_{1-x}$As semiconductor matrix. With an ab initio determined dielectric function for bismuth and well-known dielectric properties of Al${}_{x}$Ga${}_{1-x}$As solid solution, we performed calculations of the optical extinction spectra for such metamaterial using Mie’s theory. The calculations demonstrate a strong band of the optical extinction using the localized surface plasmons near a photon energy of 2.5 eV. For the semiconducting matrices with a high aluminum content x>0.7, the extinction by plasmonic nanoparticles plays the dominant role in the optical properties of the medium near the resonance photon energy.

## 1. Introduction

Most metals exhibit plasmon resonance, which originates from a collective motion of the valence electrons. In metal nanoparticles (NPs), this motion is finite and the corresponding electromagnetic field extends into the surrounding medium. It gives rise to a specific mode referred to as localized surface plasmon resonance (LSPR) [1]. In contrast to the propagating bulk and surface plasmons in extended materials, the LSPR is ready for the energy exchange with an external electromagnetic field. This exchange is especially effective near the resonant frequency, which depends on the dielectric and magnetic properties of both the NPs and the surrounding medium, as well as on the size, shape, and concentration of the NPs [2].

Metallic NPs supporting LSPR have a long history of practical use [1]. The ability to resonantly enhance the light–matter interaction is of great demand for various applications in many areas. Most important advances in the field are summarized in recent reviews [3,4,5,6]. In particular, systems in which plasmonic NPs are embedded in semiconductor materials and devices can, for instance, improve the efficiency of solar cells [7,8], substantially modify the optical properties [9,10,11], strongly enhance Raman scattering [12,13,14] and provide ultra-fast processing of qubits [15].

Fabrication of metallic NPs inside common crystalline semiconductors is a very challenging task. The fabrication technologies of Ag or Au NPs, which are materials of choice for many applications in nanoplasmonics, are not compatible with epitaxial technologies of wide industrial use, such as molecular-beam epitaxy (MBE) and vapor-phase epitaxy (VPE). Systems of Ag or Au plasmonic NPs can be deposited on the surfaces of the semiconductor nanostructures [16]. However, it is extremely hard to overgrow the Ag and Au NPs using the epitaxial semiconductor material.

While the problem of technological compatibility is currently not resolved for most metals, there is a possibility of forming nanoinclusions of group-V semimetals in the bulk of AlGaAs epitaxial films grown by MBE. The procedure is based on MBE growth at low temperature (LT), typically 150–250 °C, under As-rich conditions. These growth conditions provide a high arsenic excess, mostly in the form of arsenic antisite defects, As${}_{\mathrm{Ga}}$ [17,18]. Then, the LT-grown film is annealed at a high temperature under As over-pressure. Or it can instantly be overgrown by different epitaxial layers at a common temperature of 500–700 °C, which provides a very high crystalline quality of these layers. The high temperature activates migration of the non-stoichiometry-related point defects [19,20,21], which results in the formation of NPs composed of group-V elements. In the case of LT-grown (Al,Ga,In)As, the NPs are formed by pure arsenic [22,23,24]. However, the rather small Sb concentration (y≈0.03) in the Al${}_{x}$Ga${}_{1-x}$As${}_{1-y}$Sb${}_{y}$ systems grown at LT results in the formation of NPs with an antimony concentration ≥0.9 [25]. In the case of the LT-grown GaAs${}_{1-z}$Bi${}_{z}$ with (z≈0.02), the NPs are composed of almost pure bismuth [26,27,28]. Such NPs have a rhobohedral atomic structure and almost spherical shape, with diameters around 10–20 nm. The size and concentration of the Bi NPs in the AlGaAs matrix can be changed by varying the the Bi flax during the LT MBE, by changing the growth temperature and by post-growth heat treatments of the samples.

There were extensive investigations of the optical spectra of GaAs and Al${}_{x}$Ga${}_{1-x}$As with enbedded systems of As NPs [24,29,30]. However, no features which could be associated with LSPR were found. Systems of Sb-rich NPs exhibited noticeably strong structureless optical absorption in the range of photon energies below the band gap of Al${}_{x}$Ga${}_{1-x}$As${}_{1-y}$Sb${}_{y}$ with x≈0.3 [31,32] and x≈0.6 [33,34]. The ab initio calculations of the dielectric properties of the AsSb alloy, as well as the optical extinction spectra evaluated in the framework of the Mie theory, concluded that the Sb-rich NPs support the LSPR when embedded into the Al${}_{x}$Ga${}_{1-x}$As matrix, whereas the As-rich NPs do not [35]. The photon energy of the LSPR appeared to be just above the direct band gap of Al${}_{0.6}$Ga${}_{0.4}$As. The results of calculations were found to be consistent with the experimental observations [33].

The LSPR in the system of Bi NPs embedded into Al${}_{x}$Ga${}_{1-x}$As${}_{1-z}$Bi${}_{z}$ matrix has not been addressed so far. On the one hand, the corresponding technology is still developing, which creates difficulties for experimental investigations. On the other hand, the available data on the dielectric properties of bismuth differ substantially in the energy range of interest; see, for instance, [36,37,38,39,40], which make any theoretical predictions uncertain. Observing impressive advances in the development of diluted III-bismuthides [26,27,41,42], it seems emergent to address the LSPR problem for Bi NPs embedded in (Al,Ga)As.

In this paper, we investigate the possibility and conditions for optical observation of plasmon resonance in dilute systems of Bi nanoparticles embedded into an Al${}_{x}$Ga${}_{1-x}$As semiconductor matrix. To this end, we calculate and analyze the band structure and dielectric function of bismuth. Then, we calculate the optical extinction spectra for metamaterials composed of spherical Bi nanoparticles embedded in a Al${}_{x}$Ga${}_{1-x}$As semiconductor matrix. The calculations predict that plasmon resonance in the metamaterial should play a dominating role in the extinction of photons with energy below the direct band gap of Al${}_{x}$Ga${}_{1-x}$As semiconductor matrices with x>0.7.

## 2. Calculation Methods and Computational Details

We adopt the principles of Mie theory [43] to calculate the optical extinction for a system of Bi NPs in Al${}_{x}$Ga${}_{1-x}$As semiconductor matrix. The NPs are assumed to be spherical and randomly distributed throughout a semiconductor medium. In terms of the Mie theory, the extinction cross-section for a single NP can be represented as

(1) Cext=2πk2∑i=1∞(2i+1)Re[ai+bi].

In this equation, *k* denotes the wave vector of light in the semiconductor; $a_{i}$ and $b_{i}$ are the scattering coefficients for electric and magnetic multipoles of *i*-th order. With the assumption that the magnetic permeability is the same for the NP and surrounding matrix, the scattering coefficients can be derived using the formulas [44]

(2) $$a_{i}=\frac{m\psi_{i}\left(ms\right)\psi_{i}^{\prime }\left(s\right)-\psi_{i}\left(s\right)\psi_{i}^{\prime }\left(ms\right)}{m\psi_{i}\left(ms\right)\xi_{i}^{\prime }\left(s\right)-\xi_{i}\left(s\right)\psi_{i}^{\prime }\left(ms\right)},$$

(3) $$b_{i}=\frac{\psi_{i}\left(ms\right)\psi_{i}^{\prime }\left(s\right)-m\psi_{i}\left(s\right)\psi_{i}^{\prime }\left(ms\right)}{\psi_{i}\left(ms\right)\xi_{i}^{\prime }\left(s\right)-m\xi_{i}\left(s\right)\psi_{i}^{\prime }\left(ms\right)}.$$

Here, $m^{2}=\varepsilon /\varepsilon_{m}$ denotes the ratio of the NP and matrix permittivities; $\psi_{i}\left(\rho \right)$ and $\xi_{i}\left(\rho \right)$ are the Riccati–Bessel functions; and s=kr, where *r* is the NP radius. For a dilute system of NPs the resulting extinction coefficient, α, can be evaluated as a sum of the independent contributions from each NP.

In the case of small NPs, i.e., when its size is much smaller than the light wavelength λ, the Mie series can be represented by the electric dipole approximation. In this limit, the optical extinction is predominantly determined by absorption

(4) Cext≈Cabs=24π2r3εm3/2λIm[ε]|ε+2εm|2.

In dipole approximation, the size distribution of NPs does not influence the resulting extinction coefficient. The absorption is determined by the volume fraction, *f*, which represents the space occupied by all NPs within the metamaterial.

(5) $$\alpha =\frac{3f}{4\pi r^{3}}C_{ext}.$$

Calculation of the optical extinction using Equation (5) requires data on the dielectric permittivities of both the NPs and the Al${}_{x}$Ga${}_{1-x}$As matrix. The LSPR in the optical extinction spectra is expected near a photon energy determined by the condition $\varepsilon =-2\varepsilon_{m}$ in the dipole approximation (Equation (4)).

The dielectric permittivity of Bi, ε was calculated ab initio. The dielectric permittivity of Al${}_{x}$Ga${}_{1-x}$As was determined in terms of Adachi’s model [45].

Electronic band structure calculations were performed in the density functional theory framework employing the projector augmented-wave (PAW) method [46] implemented in the VASP code [47,48]. The Hamiltonian included the scalar relativistic corrections. The spin–orbit-coupling term was taken into account by the second variation method [49].

The atomic crystal structure was fully optimized with the use of a conjugate-gradient algorithm in order to determine the equilibrium lattice parameters and atomic positions. The structural optimization was realized with the generalized gradient approximation (GGA-PBE [50]) for the exchange-correlation energy and the DFT-D3 van der Waals (vdW) functional with Becke–Johnson damping [51]. Spin–orbit coupling was always included in the relaxation process. A force tolerance criterion of 10${}^{-4}$ eV/Å for convergence of the relaxed atomic coordinates was employed. For the total energy, the convergence criterion was 10${}^{-6}$ eV. The *k*-point mesh of 18×18×18 was used to sample the bulk Brillouin zone. To obtain the accurate bulk band structures, we adopted the modified Becke–Johnson (mBJ) semilocal exchange potential [52,53] and HSE06 screened hybrid functional [54].

The frequency-dependent dielectric matrix was calculated according to the PAW method [55] after the electronic ground state was determined within GGA-PBE and metaGGA-mBJ approaches. The local field effects are neglected in this approximation. In this case, 25×25×25 *k*-points mesh was used for the Brillouin zone sampling. The presented Bi atomic structure was visualized with vesta [56]. The Fermi surface was determined using a 25×25×25 *k*-point mesh and visualized by using FermiSurfer [57].

## 3. Calculation Results and Discussion

### 3.1. Electronic Structure and Dielectric Function of Bismuth

**Figure 1:**
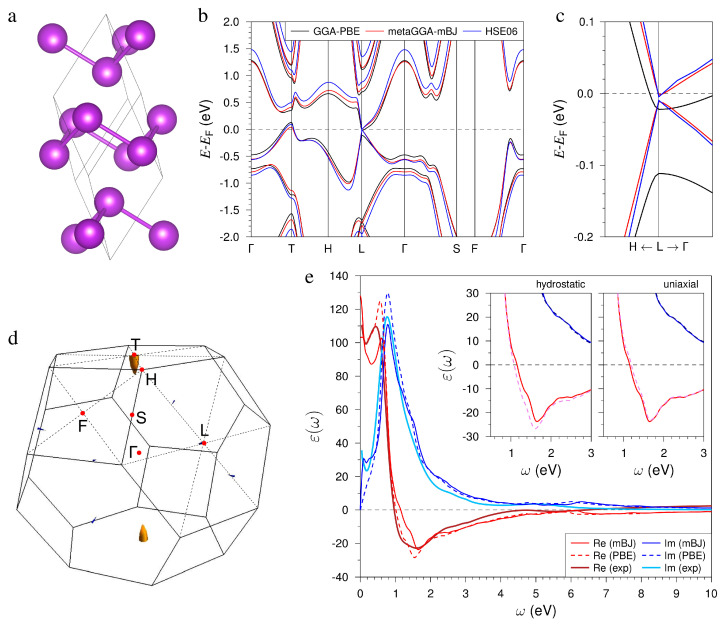
(**a**) Crystal structure of the bulk Bi. (**b**) Bulk electronic spectra calculated within three approximations: GGA-PBE, metaGGA-mBJ, and HSE06 along high symmetry lines of the bulk Brillouin zone (see panel (**d**)). (**c**) Magnified view of the spectra near the Fermi level at the L point. (**d**) Bulk Fermi surface calculated according to the GGA-PBE approach. (**e**) Imaginary and real parts of the dielectric function ε(ω) calculated within mBJ and PBE approaches in comparison with fitted experimental data from Ref. [40] (see keys for the meaning of line styles and colors). Insets show the changes in ε(ω) calculated within mBJ approach within the energy range of interest (≈1–2.5 eV) under hydrostatic (left) and uniaxial (right) strain (dashed lines) in comparison with that for equilibrium lattice (solid lines).

The bismuth possesses the A7 rhombohedral layered structure with a $R\bar{3}m$ space group and two Bi atoms per unit cell. The atomic positions within the primitive cell are ±(u,u,u). The layered structure consists of Bi bilayers (BLs) separated by van der Waals gaps (see Figure 1a). We start from the experimental crystal structure determined by the lattice parameter a=4.7459 Å and the angle $\alpha =57.237^{\circ }$, where the internal parameter u=0.237 [58]. Structure optimization within a bare GGA approach leads to elongation of the *a* parameter to 4.8648 Å and to a decrease in the rhombohedral angle to 56.8737${}^{\circ }$, whereas the GGA with the vdW corrections included demonstrates perfect agreement with experimental cell parameters (*a* and α do not change after optimization). The optimized *u* (0.233) is close to the experimental value. We use this equilibrium structure for all further calculations.

The Bi electronic structure calculated within GGA-PBE and using sophisticated semilocal mBJ and hybrid HSE06 functionals (Figure 1b) qualitatively agree with each other, demonstrating T-point hole and L-point small-electron pockets in the semimetal spectrum (see Fermi surface in Figure 1d) as well as with earlier calculations [59,60,61,62]. However, a noticeable difference between PBE and other functionals is observed at the L point (see magnified view in Figure 1c). Similar to earlier calculations, the PBE spectrum demonstrates a large 89.4 meV L-gap below the Fermi level that is a significant overestimation of the experimental L-gap (11–15 meV [63,64,65,66,67]). In contrast, our sophisticated mBJ/HSE06 calculations predict an L gap of 9.3 (mBJ) and 5.9 (HSE06) meV in good agreement with the experiment and earlier quasiparticle self-consistent GW (QSGW) [62] calculations (13 meV). Additionally, our calculations, like most earlier theoretical calculations, correctly reproduce the trivial topological phase of bismuth (calculated based on the parity eigenvalues of the energy bands below the Fermi level at time reversal invariant momentum (TRIM) points), whereas some calculations argue for nontrivial phase [68,69]. The origin of this uncertainty in the topological classification of this material is explained by the extremely small energy gap at the L TRIM point, which indicates that the system is near a topological phase transition.

Next, we examine how the accuracy in the description of the band structure affects the dielectric function. Since the metaGGA and the hybrid functional are basically consistent with each other in the description of the bulk spectrum, we will compare the results of ε(ω) calculations in the mBJ and PBE approaches.

The most relevant feature of the calculated ε reported in Figure 1e is a strong peak in Im[ε] at the energy of ∼0.8 eV. This peak originates from the interband transitions around the Γ and T points of the Brillouin zone [38,59]. The amplitude of this peak is about 130 in the PBE calculation, whereas in the mBJ it is about 110, which is very close to the experimental value of ∼115 obtained in the spectroscopic ellipsometry measurements [40]. The differences in the PBE and the mBJ band structures are reflected in the shapes of the absorption peak evaluated in both approximations. Especially notable differences in Im[ε] in two calculating curves can be detected at energies below 0.4 eV, which can be linked to the different Fermi surfaces around the *T* and *L* points [38,59]. Again, in this energy region, the mBJ calculated curve better fits the experimental data.

The presence of this dominating peak in Im[ε] is reflected in a remarkable behavior of Re[ε], which becomes negative at energies above 0.95 eV in the calculation with the employment of the mBJ band structure. This value is very close to the experimental observations [40]. In the PBE calculation, Re[ε] becomes negative at the energy of 1.2 eV. After crossing the zero line, the PBE (mBJ) curve for Re[ε] reaches a minimum value of −28.5 (−23.6) at ω = 1.55 eV (1.65 eV). In the same region, a shallow minimum is observed in the experimental Re[ε] curve [40] as well. Due to a small number of free carriers at the Bi Fermi surfaces, the Drude peaks in Im[ε] in both calculated curves are very small. In consequence, the real part of the calculated dielectric functions becomes positive at low energies, maintaining its value above 80. The large value of Re[ε] below ∼0.6 eV is observed in the experiment as well [40]. However, the calculations do not reproduce a drop in the experimental Re[ε] down to 30 at energies below 0.05 eV. We attribute this discrepancy to the fact that the experiment was realized at a finite temperature, whereas the calculations are realized at zero temperature. Subsequently, the number of free carriers may be different.

The Bi NPs embedded into the crystalline Al${}_{x}$Ga${}_{1-x}$As matrix can be mechanically stressed. If the system of the Bi NPs is formed by a self-organization process directly in the bulk of the matrix, the Bi atoms replace the corresponding As, Ga, and Al atoms in the host lattice. Due to a difference in the atomic volumes, this replacement should create a compression in the interior of the Bi nanoinclusions. Since the volume difference between Bi atoms and replaced host atoms is quite large, one could expect very strong values of the compressing stress and corresponding elastic strain, which, in turn, may lead to strong variations in the dielectric function [70,71]. Such highly stressed states of NPs are, however, unstable against relaxation phenomena; for instance, against the formation of satellite dislocation loops [72]. While most of the elastic energy has to be released due to relaxation, some residual strain remains persistent, since the formation of dislocations is associated with a certain threshold. Our estimations show that Bi nanoparticles embedded in a semiconductor matrix can experience a small compressive strain, which does not exceed 0.5%. Another type of possible elastic deformation originates from the fact that the atomic structure of Bi NPs is rhombohedral, whereas the Al${}_{x}$Ga${}_{1-x}$As matrix possesses a cubic zincblende structure. As a result, one can expect a deviatoric deformation composed of some compression or stretch along the trigonal axes with an opposite sign of deformation in the perpendicular plane. In this regard, we considered the variations in the electronic structure and the dielectric function caused by small deformations of the atomic lattice. To this end, we simulated both a hydrostatic compression, when the lattice parameters are reduced by 0.5% at fixed α, which is the upper estimate of possible deformations; and a volume-conserving uniaxial deformation, which is when the $a_{\mathrm{hex}}$ lattice parameter in the Bi-BL plane is reduced by the same 0.5% while $c_{\mathrm{hex}}$ is enlarged by ≈1% to keep the cell volume unchanged. The latter type of deformation is equivalent to varying the rhombohedral angle α with a fixed rhombohedral lattice parameter. At both hydrostatic and uniaxial cell deformations, the atomic positions were allowed to relax. In the case of hydrostatic compression, the interlayer distance in the bilayer increased by 0.88% compared to the equilibrium one, and the vdW spacing decreased by 1.48%. In the case of uniaxial deformation, both BL and vdW spacings increased by 1.18% and 0.89%, respectively.

When hydrostatic compression is applied, the L-gap in the mBJ-derived spectrum increases to 20.7 meV and the band topology remains trivial. For uniaxial deformation, earlier accurate QSGW calculations have predicted the topological phase transition at decreasing $a_{\mathrm{hex}}$ by 0.4% [62]. In our mBJ calculation for the structure with $a_{\mathrm{hex}}$ reduced by 0.5%, the L gap amounts to 1.6 meV, and according to the wave functions parity calculations, the $Z_{2}$ topological invariant becomes equal to 1, i.e., uniaxially strained Bi occurs in the topological phase while remaining semimetal. The changes in ε(ω) under both types of deformations are shown in the insets in Figure 1e. As can be seen in the left inset, the small hydrostatic compression results in a shift of the minimum in the real part to 1.516 eV (where it reaches −30.615), while uniaxial stress has a negligible effect on the real part of ε. At the same time, there are no significant changes in the imaginary parts at both types of deformations.

### 3.2. Electronic Structure and Dielectric Function of Al${}_{x}$Ga${}_{1-x}$As

The Al${}_{x}$Ga${}_{1-x}$As semiconductor solid solutions possess the zinc-blended atomic structure with $F\bar{4}3m$ space group. Their electronic band structure is well documented [45]. The valence band consists of three sub-bands with maxima at the Γ point of the Brillouin zone. The valence-band top is degenerated by the heavy- and the light-hole sub-bands. The third sub-band is split down due to the spin–orbit interaction. The splitting, Δ, depends on the aluminum concentration, *x*, as follows

(6) Δ=(0.34−0.04x)eV.

The conduction band minima are situated at Γ, *X*, and *L* points of the Brillouin zone. At room temperature, the direct and indirect band gaps are described as a function of the aluminum concentration according to the following relations [73]

(7) $$E_{g}^{\Gamma }=\left(1.424+1.155x+0.37x^{2}\right)\mathrm{eV},$$

(8) $$E_{g}^{X}=\left(1.9+0.124x+0.144x^{2}\right)\mathrm{eV},$$

(9) $$E_{g}^{L}=\left(1.71+0.69x\right)\mathrm{eV}.$$

It follows that the Al${}_{x}$Ga${}_{1-x}$As semiconductor solid solution has a direct band gap at the Γ point at x<0.41. For AlAs and Al-rich solid solutions, the lowest band gap is indirect.

The dielectric properties of the Al${}_{x}$Ga${}_{1-x}$As semiconductor solid solutions have been extensively studied [45,74,75,76]. In the energy range of interest (from 0.5 to 5 eV), the dielectric function is determined by the band structure with major contributions from the critical points in the joint density of states within this interval [74,75,76]. The contribution of the Wannier excitons to the dielectric function was addressed in [77].

In this paper, we utilized a semi-empirical model developed by S. Adachi [45]. This model provided a relatively simple-to-calculate, reliable equation for the real part of the dielectric function below the direct band edge, $E_{g}^{\Gamma }$

(10) $$\varepsilon \left(\omega \right)=A\{f\left(\chi \right)+0.5{\left[E_{g}^{\Gamma }/\left(E_{g}^{\Gamma }+\Delta \right)\right]}^{3/2}f\left(\chi_{SO}\right)\}+B,$$

(11) $$f\left(\chi \right)=\chi^{-2}\left[2-{\left(1+\chi \right)}^{1/2}-{\left(1-\chi \right)}^{1/2}\right],$$

(12) $$\chi =\hbar \omega /E_{g}^{\Gamma },$$

(13) $$\chi_{SO}=\hbar \omega /\left(E_{g}^{\Gamma }+\Delta \right),$$

where ℏω is the photon energy; *A* and *B* are parameters. Two terms in the curly brackets in Equation (10) represent free electron–hole pair and exciton contributions related to the $E_{g}^{\Gamma }$ and $E_{g}^{\Gamma }+\Delta$ gaps. The *B* parameter corresponds to the background dielectric permittivity provided by the higher-lying-gap transitions such as $E_{1}$, $E_{1}+\Delta_{1}$, $E_{2}$. Composition-dependent representations of the *A* and *B* parameters are deduced from experimental data, as follows

(14) A(x)=6.3+19.0x,

(15) B(x)=9.4−10.2x.

At energies exceeding a value of direct band gap, the matrix dielectric permittivity is constant.

The imaginary part of the dielectric function for the Al${}_{x}$Ga${}_{1-x}$As semiconductor solid solutions can be calculated by using Kramers–Kronig relations. Its value appears to be much smaller than that of Bi in the whole energy range of interest (from 0.5 to 5 eV). Therefore, the dielectric function of the semiconductor matrix is assumed to be real in our calculations of the optical properties of the system of the Bi NPs embedded into Al${}_{x}$Ga${}_{1-x}$As.

### 3.3. Localized Surface Plasmons

**Figure 2:**
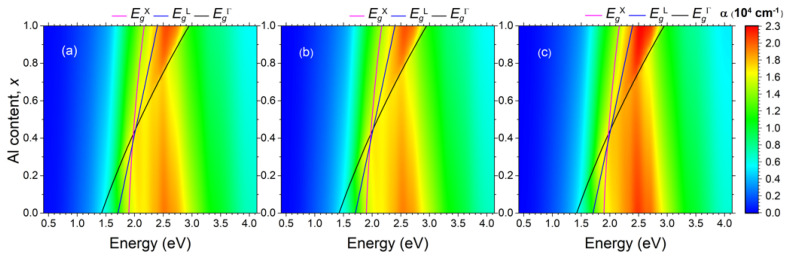
Maps of the optical extinction spectra for systems of spherical Bi NPs embedded into Al${}_{x}$Ga${}_{1-x}$As semiconductor matrices of different chemical composition *x*. Solid lines mark the edges of the direct ($E_{g}^{\Gamma }$) and indirect ($E_{g}^{X}$, $E_{g}^{L}$) band gaps. The diameter of NPs is 10 nm. The filling factor is 1%. The Bi NPs are (**a**) mechanically relaxed, (**b**) stretched by 1% along trigonal axes and compressed by 0.5% in the perpendicular plane, and (**c**) compressed by 0.5% in all the directions.

Figure 2 presents calculated maps of the optical extinction spectra for systems of spherical Bi NPs embedded in the semiconductor Al${}_{x}$Ga${}_{1-x}$As matrix. The spectra were obtained by using the Mie theory via the formalism described in Section 2. Computations have been performed for the whole range of Al content, *x*, from 0 to 1. The dielectric properties of the semiconductor matrix were calculated by employing the above-mentioned Adachi model (Section 3.2), while the dielectric permittivity of the Bi NPs was obtained within DFT approach (metaGGA-mBJ) outlined in Section 3.1. Keeping in mind the data of structural investigations [26,27,28], the NPs’ diameter and their volume fraction have been set to 10 nm and 1%, respectively. Solid lines at the optical extinction map show the dependencies of the edges of the direct ($E_{g}^{\Gamma }$) and indirect ($E_{g}^{X}$, $E_{g}^{L}$) band gaps according to Equations (7)–(9).

The map of the optical extinction in Figure 2a corresponds to mechanically relaxed NPs. The LSPR manifests itself as a strong optical extinction in the vicinity of 2.5 eV. For the Al${}_{x}$Ga${}_{1-x}$As matrix with relatively low Al content x<0.6, the LSPR occurs within the fundamental absorption band governed by the direct optical transition above the Γ point. For the Al-rich matrix, the LSPR is above the indirect band gap, but below the $E_{g}^{\Gamma }$.

It is evident that even a dilute system of Bi NPs has a substantial impact on the optical properties of the host Al${}_{x}$Ga${}_{1-x}$As matrix, especially the Al-rich one. In fact, the optical extinction coefficient provided by the LSPR is higher than $2\times 10^{4}$ cm${}^{-1}$, whereas the optical absorption coefficient in the Al${}_{x}$Ga${}_{1-x}$As due to indirect transitions is less than $1\times 10^{3}$ cm${}^{-1}$ [78]. In the case of direct optical transitions, the absorption coefficient in the semiconductor matrix ranges from $1\times 10^{4}$ to $1\times 10^{5}$ cm${}^{-1}$ [78]. Even in this case, the influence of the LSPR can be substantial.

**Figure 3:**
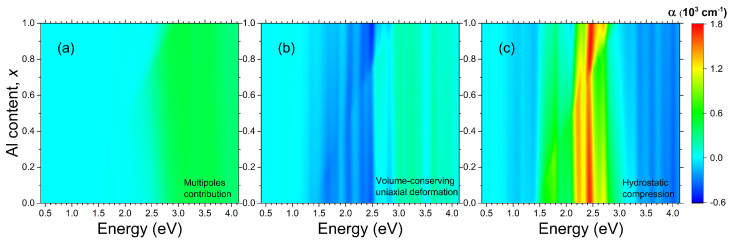
Maps of the differential optical extinction spectra for systems of spherical Bi NPs embedded into Al${}_{x}$Ga${}_{1-x}$As semiconductor matrices of different chemical composition *x*. The diameter of NPs is 10 nm. The filling factor is 1%. (**a**) multipole contributions, (**b**) volume-conserving uniaxial deformation, (**c**) hydrostatic deformation.

Figure 2c represents the map of the optical extinction for a system of Bi NPs, which are compressed hydrostatically by 0.5% in all the lattice parameters. For better visibility, the changes in the extinction spectra for this type of deformation are plotted in Figure 3c. It is evident that the hydrostatic deformation enhances the extinction in the resonant band by 5–10%. Figure 2b represents the map of the optical extinction for a system of Bi NPs, which are compressed in plane by 0.5% and stretched out-of-plane by 1%, so that there are no changes in the volume. This kind of strain appears to slightly reduce the resonant extinction, as clearly demonstrated in Figure 3b. Thus, the hydrostatic compression, keeping the topologically trivial phase of bismuth, results in noticeable enhancement in resonant extinction, whereas the topological phase transition taking place under uniaxial deformation, where spin-polarized states should arise at the nanoparticle–matrix interface, has only a small effect on the optical properties, and has the opposite effect, leading to a decrease in resonant extinction.

The LSPR energy depends on the NP size [43]. For small NPs, r≪λ, it is mostly determined by the electric dipole mode. This inequality is well satisfied in the whole energy range of interest for the Bi NPs with a diameter of 10 nm, which we consider in our calculations. It is evident from Figure 3a that all the multipole modes make a negligible contribution to the total optical extinction for the photon energy at and below 2.5 eV. This multipole-related portion of the optical extinction near the plasmonic peak (2.5 eV) is equal to several tens of cm${}^{-1}$ out of a peak total value of 21,000 cm${}^{-1}$. For the photon energy above the LSPR, the contribution of multipoles reaches several hundreds of cm${}^{-1}$. So, the dipole approximation appears to be valid for an accurate description of the Bi NPs with diameters ranging from several nm to several tens of nm. This range of sizes is typical for the compound metamaterials with systems of the Bi NPs formed by self-organization in the Al${}_{x}$Ga${}_{1-x}$As matrix [26]. An important consequence of the prevalence of the dipole mode is that the cumulative response of a system of the Bi NPs embedded into the Al${}_{x}$Ga${}_{1-x}$As matrix does not depend on their actual size distribution. The position and shape of the LSPR can be determined from Equation (4) and the magnitude of the resonance in a dilute system of the Bi NPs is solely determined by the total volume occupied by the NPs (see Equation (5)).

Since the diameter of the Bi NP is as small as 10 nm, a question arises regarding the applicability of the classical electrodynamics in terms of the Mie theory to calculation of the LSPR. It should be noted that the LSPR mode considered in this paper does not travel; it is localized at the interface of the Bi NP and surrounding semiconductor matrix. Therefore, the quantum confinement affects the LSPR via variations of the Bi permittivity due to changes in the electronic band structure. These variations should be minor in the energy range of interest for the Bi NPs with diameter of 10 nm even if the quantum confinement strongly changes the band structure near the Fermi surface where the conduction and valence bands overlap just slightly as demonstrated in Figure 1b–d. This statement is supported by our calculations of the dielectric function under the mechanical stresses, which alters the Bi band structure at the Fermi level and even leads to change the topology of the bands. However, it is evident from Figure 2 that the resulting changes in the LSPR-related optical extinction are minor. Note that the colored scale of the corresponding differential spectra in Figure 3 is substantially expanded for visibility of the phenomenon.

The effect of the quantum confinement of the LSPR was directly addressed in Ref. [79] for Ag NPs. Both experimental investigations and ab initio DFT calculations showed a blueshift of the resonance for NPs below 10 nm because the permittivity of Ag was altered by the quantum size effect. For NPs with diameters of 10 nm and larger, the calculations based on classical electrodynamics with bulk dielectric permittivity appeared to be quite accurate.

Spatial confinement has different impacts on plasmonic modes of different types. In fact, the transformation of the semimetal to a semiconductor [27] and changes in the band topology should drastically change the plasmon resonances related to the electrons near the Fermi surface. These phenomena could influence the optical properties of the medium at the low-energy end $\hbar \omega \ll E_{\mathrm{LSPR}}$. At the opposite side of the energy spectrum of Bi, there is a bulk plasmon resonance, the energy of which increases from 13.8 eV for the particles of 500 nm in diameter to 28.2 eV for the NPs of 5 nm [80]. These energies are well above the considered LSPR situated near 2.5 eV. It should be noted that the bulk plasmon mode does not interact with light [1].

It is worth noting that with an increase in Al content, the fundamental absorption edge of the Al${}_{x}$Ga${}_{1-x}$As matrix is shifted to higher energies, widening the optical transparency window of the material. Hence, a high Al concentration makes it potentially possible to experimentally observe the LSPR resonance peak in the optical absorption spectra. Our modeling shows that for the Al${}_{x}$Ga${}_{1-x}$As compound with x>0.7, the LSPR resonance peak can be resolved in the optical absorption spectra. So, the metamaterials based on the Al-rich Al${}_{x}$Ga${}_{1-x}$As semiconductor matrix with built-in systems of Bi NPs should demonstrate a strong and ultrafast optical response in a wide band of the photon energies near 2.5 eV. This response is in drastic contrast to the optical properties of the conventional Al-rich Al${}_{x}$Ga${}_{1-x}$As. This material with an indirect band gap demonstrates quite a weak optical absorption and a long radiative lifetime of optically excited non-equilibrium electrons.

## 4. Conclusions

We propose a new optical metamaterial based on a system of Bi NPs embedded in the Al${}_{x}$Ga${}_{1-x}$As matrix. Analysis of the dielectric properties of the Bi NPs shows that they can support the surface plasmon resonance in a dielectric medium in a spectral range from 1 to 5 eV. Being embedded in the semiconducting Al${}_{x}$Ga${}_{1-x}$As matrix, they ensure the LSPR is near an energy level of 2.5 eV. Even a dilute system of Bi NPs provides a strong resonant optical absorption, which should play a dominating role in the optical properties of the medium when the aluminum content *x* in the Al${}_{x}$Ga${}_{1-x}$As solid solution is higher than 0.7. Formation of the proposed metamaterial seems to be feasible via self-organization of Bi NPs in epitaxial Al${}_{x}$Ga${}_{1-x}$As films supersaturated by bismuth. The required LT MBE technology is compatible with the conventional growth procedure utilized for the fabrication of Al${}_{x}$Ga${}_{1-x}$As-based electronic and optoelectronic devices.

## Data Availability

Data is contained within the article.

## Abbreviations

The following abbreviations are used in this manuscript:

LSPR	Localized surface plasmon resonance.
NP	Nanoparticle.
MBE	Molecular-beam epitaxy.
VPE	Vapor-phase epitaxy.
LT	Low temperature.
PAW	Projector-augmented wave.
GGA-PBE	Perdew–Burke–Ernzerhof generalized gradient approximation.
vdW	van der Waals.
BL	Bilayer.
DFT-D3	vdW functional with Becke–Johnson damping.
mBJ	modified Becke–Johnson semilocal exchange potential.
HSE06	Heyd–Scuseria–Ernzerhof screened hybrid functional.
QSGW	Quasiparticle self-consistent GW.
TRIM	Time reversal-invariant momentum.

## References

[1] Maier S. (2007). Plasmonics: Fundamentals and Applications.

[2] Garcia M.A. (2011). Surface plasmons in metallic nanoparticles: Fundamentals and applications. J. Phys. D Appl. Phys..

[3] Wang L., Kafshgari M.H., Meunier M. (2020). Optical Properties and Applications of Plasmonic-Metal Nanoparticles. Adv. Funct. Mater..

[4] Rivera N., Kaminer I. (2020). Light–matter interactions with photonic quasiparticles. Nature Rev. Phys..

[5] Koya A.N., Romanelli M., Kuttruff J., Henriksson N., Stefancu A., Grinblat G., De Andres A., Schnur F., Vanzan M., Marsili M. (2023). Advances in ultrafast plasmonics. Appl. Phys. Rev..

[6] Babicheva V.E. (2023). Optical Processes behind Plasmonic Applications. Nanomaterials.

[7] Mandal P. (2022). Application of Plasmonics in Solar Cell Efficiency Improvement: A Brief Review on Recent Progress. Plasmonics.

[8] Ibrahim Zamkoye I., Lucas B., Vedraine S. (2023). Synergistic Effects of Localized Surface Plasmon Resonance, Surface Plasmon Polariton, and Waveguide Plasmonic Resonance on the Same Material: A Promising Hypothesis to Enhance Organic Solar Cell Efficiency. Nanomaterials.

[9] Agiotis L., Meunier M. (2022). Nonlinear Propagation of Laser Light in Plasmonic Nanocomposites. Laser Photonics Rev..

[10] Rajamanikandan R., Sasikumar K., Kosame S., Ju H. (2023). Optical Sensing of Toxic Cyanide Anions Using Noble Metal Nanomaterials. Nanomaterials.

[11] Zhang C., Huang C., Pu M., Song J., Zhao Z., Wu X., Luo X. (2017). Dual-band wide-angle metamaterial perfect absorber based on the combination of localized surface plasmon resonance and Helmholtz resonance. Sci. Rep..

[12] Fleischmann M., Hendra P., McQuillan A. (1974). Raman spectra of pyridine adsorbed at a silver electrode. Chem. Phys. Lett..

[13] Bai S., Ren X., Obata K., Ito Y., Sugioka K. (2022). Label-free trace detection of bio-molecules by liquid-interface assisted surface-enhanced Raman scattering using a microfluidic chip. Opto-Electron. Adv..

[14] Wang Y., Xu X., Li Y., Li C., Wang X., Wu J., Li Y. (2024). Handcrafted silver substrates boost surface plasmon resonance for ultra-sensitive lipid analysis. Talanta.

[15] Bogdanov S., Boltasseva A., Shalaev V. (2019). Overcoming quantum decoherence with plasmonics. Science.

[16] Toropov N.A., Gladskikh I.A., Gladskikh P.V., Kosarev A.N., Preobrazhenskiĭ V.V., Putyato M.A., Semyagin B.R., Chaldyshev V.V., Vartanyan T.A. (2017). Absorption and photoluminescence of epitaxial quantum dots in the near field of silver nanostructures. J. Opt. Technol..

[17] Liu X., Prasad A., Nishio J., Weber E.R., Liliental-Weber Z., Walukiewicz W. (1995). Native point defects in low-temperature-grown GaAs. Appl. Phys. Lett..

[18] Lavrent’eva L.G., Vilisova M.D., Preobrazhenskii V.V., Chaldyshev V.V. (2002). Low-temperature molecular beam epitaxy of GaAs: Influence of crystallization conditions on structure and properties of layers. Crystallogr. Rep..

[19] Geursen R., Lahiri I., Dinu M., Melloch M.R., Nolte D.D. (1999). Transient enhanced intermixing of arsenic-rich nonstoichiometric AlAs/GaAs quantum wells. Phys. Rev. B.

[20] Bert N.A., Chaldyshev V.V., Musikhin Y.G., Suvorova A.A., Preobrazhenskii V.V., Putyato M.A., Semyagin B.R., Werner P. (1999). In–Ga intermixing in low-temperature grown GaAs delta doped with In. Appl. Phys. Lett..

[21] Chaldyshev V.V., Bert N.A., Musikhin Y.G., Suvorova A.A., Preobrazhenskii V.V., Putyato M.A., Semyagin B.R., Werner P., Gösele U. (2001). Enhanced As–Sb intermixing of GaSb monolayer superlattices in low-temperature grown GaAs. Appl. Phys. Lett..

[22] Warren A.C., Woodall J.M., Freeouf J.L., Grischkowsky D., McInturff D.T., Melloch M.R., Otsuka N. (1990). Arsenic precipitates and the semi-insulating properties of GaAs buffer layers grown by low-temperature molecular beam epitaxy. Appl. Phys. Lett..

[23] Claverie A., Liliental-Weber Z. (1992). Structure and orientation of As precipitates in GaAs grown at low temperature by molecular beam epitaxy. Philos. Mag. A.

[24] Bert N.A., Veinger A.I., Vilisova M.D., Goloshchapov S.I., Ivonin I.V., Kozyrev S.V., Kunitsyn A.E., Lavrent’eva L.G., Lubyshev D.I., Preobrazhenskii V.V. (1993). Gallium arsenide grown by molecular beam epitaxy at low temperatures: Crystal structure, properties, superconductivity. Phys. Solid State.

[25] Bert N.A., Chaldyshev V.V., Cherkashin N.A., Nevedomskiy V.N., Preobrazhenskii V.V., Putyato M.A., Semyagin B.R., Ushanov V.I., Yagovkina M.A. (2019). Metallic AsSb nanoinclusions strongly enriched by Sb in AlGaAsSb metamaterial. J. Appl. Phys..

[26] Wu M., Luna E., Puustinen J., Guina M., Trampert A. (2014). Formation and phase transformation of Bi-containing QD-like clusters in annealed GaAsBi. Nanotechnology.

[27] Butkutė R., Niaura G., Pozingytė E., Čechavičius B., Selskis A., Skapas M., Karpus V., Krotkus A. (2017). Bismuth Quantum Dots in Annealed GaAsBi/AlAs Quantum Wells. Nanoscale Res. Lett..

[28] Baladés N., Sales D., Herrera M., Tan C., Liu Y., Richards R., Molina S. (2018). Analysis of Bi distribution in epitaxial GaAsBi by aberration-corrected HAADF-STEM. Nanoscale Res. Lett..

[29] Nolte D.D. (1994). Optical scattering and absorption by metal nanoclusters in GaAs. J. Appl. Phys..

[30] Dankowski S.U., Streb D., Ruff M., Kiesel P., Kneissl M., Knüpfer B., Döhler G.H., Keil U.D., Sorenson C.B., Verma A.K. (1996). Above band gap absorption spectra of the arsenic antisite defect in low temperature grown GaAs and AlGaAs. Appl. Phys. Lett..

[31] Ushanov V.I., Chaldyshev V.V., Il’inskaya N.D., Lebedeva N.M., Yagovkina M.A., Preobrazhenskii V.V., Putyato M.A., Semyagin B.R. (2014). Fröhlich resonance in the AsSb/AlGaAs system. Phys. Solid State.

[32] Ushanov V.I., Chaldyshev V.V., Bert N.A., Nevedomsky V.N., Il’inskaya N.D., Lebedeva N.M., Preobrazhenskii V.V., Putyato M.A., Semyagin B.R. (2015). Plasmon resonance in new AsSb–AlGaAs metal–semiconductor metamaterials. Semiconductors.

[33] Bert N., Ushanov V., Snigirev L., Kirilenko D., Ulin V., Yagovkina M., Preobrazhenskii V., Putyato M., Semyagin B., Kasatkin I. (2022). Metal-Semiconductor AsSb-Al_0.6_Ga_0.4_As_0.97_Sb_0.03_ Metamaterial. Materials.

[34] Snigirev L., Ushanov V., Ivanov A., Bert N., Kirilenko D., Yagovkina M., Preobrazhenskii V., Putyato M., Semyagin B., Kasatkin I. (2023). Structure and optical properties of a composite AsSb-Al_0.6_Ga_0.4_As_0.97_Sb_0.03_ Metamaterial. Semiconductors.

[35] Silkin V.M., Eremeev S.V., Ushanov V.I., Chaldyshev V.V. (2023). Localized Surface Plasmon Resonance in Metamaterials Composed of As_1−*z*_Sb_*z*_ Semimetal Nanoparticles in Al_*x*_Ga_1−*x*_As_1−*y*_Sb_*y*_ Semiconductor Matrix. Nanomaterials.

[36] Cardona M., Greenaway D.L. (1964). Optical Properties and Band Structure of Group IV-VI and Group V Materials. Phys. Rev..

[37] Lenham A.P., Treherne D.M., Metcalfe R.J. (1965). Optical Properties of Antimony and Bismuth Crystals. J. Opt. Soc. Am..

[38] Hunderi O. (1975). Optical properties of crystalline and amorphous bismuth films. J. Phys. F Metal Phys..

[39] Werner W.S.M., Glantschnig K., Ambrosch-Draxl C. (2009). Optical Constants and Inelastic Electron-Scattering Data for 17 Elemental Metals. J. Phys. Chem. Ref. Data.

[40] Toudert J., Serna R., Camps I., Wojcik J., Mascher P., Rebollar E., Ezquerra T.A. (2017). Unveiling the Far Infrared-to-Ultraviolet Optical Properties of Bismuth for Applications in Plasmonics and Nanophotonics. J. Phys. Chem. C.

[41] Wang L., Zhang L., Yue L., Liang D., Chen X., Li Y., Lu P., Shao J., Wang S. (2017). Novel Dilute Bismide, Epitaxy, Physical Properties and Device Application. Crystals.

[42] Richards R.D., Bailey N.J., Liu Y., Rockett T.B.O., Mohmad A.R. (2022). GaAsBi: From Molecular Beam Epitaxy Growth to Devices. Phys. Status Solidi.

[43] Mie G. (1908). Beiträge zur Optik trüber Medien, speziell kolloidaler Metallösungen. Ann. Phys..

[44] Bohren C.F., Huffman D.R. (2008). Absorption and Scattering of Light by Small Particles.

[45] Adachi S. (1985). GaAs, AlAs and Al_*x*_Ga_1−*x*_As: Material parameters for use in research and device applications. J. Appl. Phys..

[46] Blöchl P.E. (1994). Projector augmented-wave method. Phys. Rev. B.

[47] Kresse G., Furthmüller J. (1996). Efficient iterative schemes for ab initio total-energy calculations using a plane-wave basis set. Phys. Rev. B.

[48] Kresse G., Joubert D. (1999). From ultrasoft pseudopotentials to the projector augmented-wave method. Phys. Rev. B.

[49] Koelling D.D., Harmon B.N. (1977). A technique for relativistic spin-polarised calculations. J. Phys. C Solid State Phys..

[50] Perdew J.P., Burke K., Ernzerhof M. (1996). Generalized Gradient Approximation Made Simple. Phys. Rev. Lett..

[51] Grimme S., Ehrlich S., Goerigk L. (2011). Effect of the damping function in dispersion corrected density functional theory. J. Comp. Chem..

[52] Becke A.D., Johnson E.R. (2006). A simple effective potential for exchange. J. Chem. Phys..

[53] Tran F., Blaha P. (2009). Accurate Band Gaps of Semiconductors and Insulators with a Semilocal Exchange-Correlation Potential. Phys. Rev. Lett..

[54] Krukau A.V., Vydrov O.A., Izmaylov A.F., Scuseria G.E. (2006). Influence of the exchange screening parameter on the performance of screened hybrid functionals. J. Chem. Phys..

[55] Gajdoš M., Hummer K., Kresse G., Furthmüller J., Bechstedt F. (2006). Linear optical properties in the projector-augmented wave methodology. Phys. Rev. B.

[56] Momma K., Izumi F. (2011). *VESTA 3* for three-dimensional visualization of crystal, volumetric and morphology data. J. Appl. Crystallogr..

[57] Kawamura M. (2019). FermiSurfer: Fermi-surface viewer providing multiple representation schemes. Comp. Phys. Commun..

[58] Wyckoff R.W.G. (1963). Crystal Structures.

[59] Liu Y., Allen R.E. (1995). Electronic structure of the semimetals Bi and Sb. Phys. Rev. B.

[60] Fu L., Kane C.L. (2007). Topological insulators with inversion symmetry. Phys. Rev. B.

[61] Zhang H.J., Liu C.X., Qi X.L., Deng X.Y., Dai X., Zhang S.C., Fang Z. (2009). Electronic structures and surface states of the topological insulator Bi_1−*x*_Sb_*x*_. Phys. Rev. B.

[62] Aguilera I., Friedrich C., Blügel S. (2015). Electronic phase transitions of bismuth under strain from relativistic self-consistent *GW* calculations. Phys. Rev. B.

[63] Maltz M., Dresselhaus M.S. (1970). Magnetoreflection Studies in Bismuth. Phys. Rev. B.

[64] Vecchi M.P., Dresselhaus M.S. (1974). Temperature dependence of the band parameters of bismuth. Phys. Rev. B.

[65] Isaacson R.T., Williams G.A. (1969). Alfvén-Wave Propagation in Solid-Stae Plasmas. III. Quantum Oscillations of the Fermi Surface of Bismuth. Phys. Rev..

[66] Brown R.N., Mavroides J.G., Lax B. (1963). Magnetoreflection in Bismuth. Phys. Rev..

[67] Smith G.E., Baraff G.A., Rowell J.M. (1964). Effective *g* Factor of Electrons and Holes in Bismuth. Phys. Rev..

[68] Ohtsubo Y., Perfetti L., Goerbig M.O., Fèvre P.L., Bertran F., Taleb-Ibrahimi A. (2013). Non-trivial surface-band dispersion on Bi(111). New J. Phys..

[69] Ohtsubo Y., Kimura S.i. (2016). Topological phase transition of single-crystal Bi based on empirical tight-binding calculations. New J. Phys..

[70] Rodriguez-Prieto A., Bergara A., Silkin V.M., Echenique P.M. (2006). Complexity and Fermi surface deformation in compressed lithium. Phys. Rev. B.

[71] Errea I., Rousseau B., Eiguren A., Bergara A. (2012). Optical properties of calcium under pressure from first-principles calculations. Phys. Rev. B.

[72] Chaldyshev V.V., Bert N.A., Kolesnikova A.L., Romanov A.E. (2009). Stress relaxation scenario for buried quantum dots. Phys. Rev. B.

[73] Ioffe Institute NSM Archive: Band Structure and Carrier Concentration of AlGaAs. https://www.ioffe.ru/SVA/NSM/Semicond/AlGaAs/bandstr.html.

[74] Jenkins D.W. (1990). Optical constants of Al_*x*_Ga_1−*x*_As. J. Appl. Phys..

[75] Djurišić A.B., Rakić A.D., Kwok P.C.K., Li E.H., Majewski M.L., Elazar J.M. (1999). Modeling the optical constants of Al_*x*_Ga_1−*x*_As alloys. J. Appl. Phys..

[76] Lin C.H., Meese J. (1993). Optical properties of bulk Al_*x*_Ga_1−*x*_As. J. Appl. Phys..

[77] Tanguy C. (1995). Optical dispersion by Wannier excitons. Phys. Rev. Lett..

[78] Adachi S. (1989). Optical dispersion relations for GaP, GaAs, GaSb, InP, InAs, InSb, Al_*x*_Ga_1−*x*_As, and In_1−*x*_Ga_*x*_As_*y*_P_1−*y*_. J. Appl. Phys..

[79] Scholl J.A., Koh A.L., Dionne J.A. (2012). Quantum plasmon resonances of individual metallic nanoparticles. Nature.

[80] Wang Y.W., Kim J.S., Kim G.H., Kim K.S. (2006). Quantum size effects in the volume plasmon excitation of bismuth nanoparticles investigated by electron energy loss spectroscopy. Appl. Phys. Lett..

